# Doxycycline Inducible Chimeric Antigen Receptor T Cells Targeting CD147 for Hepatocellular Carcinoma Therapy

**DOI:** 10.3389/fcell.2019.00233

**Published:** 2019-10-11

**Authors:** Ren-Yu Zhang, Ding Wei, Ze-Kun Liu, Yu-Le Yong, Wei Wei, Zhi-Yun Zhang, Jian-Jun Lv, Zhao Zhang, Zhi-Nan Chen, Huijie Bian

**Affiliations:** Department of Cell Biology, National Translational Science Center for Molecular Medicine, Fourth Military Medical University, Xi’an, China

**Keywords:** chimeric antigen receptor, CD147, hepatocellular carcinoma, tetracycline-inducible systems, immunotherapy

## Abstract

Chimeric antigen receptor T cell (CAR-T) therapy to hematological malignancies has demonstrated tremendous clinical outcomes. However, the therapeutic efficacy of CAR-T cells in solid tumors remains limited due to the scarcity of tumor-specific antigen targets and the poor infiltration of CAR-T cells into tumor tissue. In this study, we developed a novel inducible CAR-T cell system which targets CD147, a tumor-associated antigen for hepatocellular carcinoma (HCC). To minimize potential toxicities of CAR-T cell therapy, the Tet-On 3G system was introduced to induce CD147CAR expression in the right place at the right time. Specifically, Tet-CD147CAR lentiviral vector (LV-Tet-CD147CAR) was constructed, which comprised CD147CAR controlled by the Tet-On system. Tet-CD147CART cells were successfully generated from activated T cells by infection with LV-Tet-CD147CAR. Proliferation, cytotoxicity, and cytokine secretion of Tet-CD147CART cells were significantly increased against CD147-positive cancer cells in the presence of doxycycline (Dox) compared to Tet-CD147CART cells in the absence of Dox and PBMCs. Consistently, *in vivo* studies indicated that the tumor growth in nude mice was significantly inhibited by (Dox+) Tet-CD147CART cells through multiple intratumoral administration. Taken together, our results indicated that the expression and activity of CD147CAR were controlled by Dox both *in vitro* and *in vivo*, which facilitated decreased toxicity and adverse effects to CAR-T cell therapy. Moreover, this study provides viable evidence in support of the potential benefits and translation of this strategy of CAR-T cells targeting CD147 for the treatment of patients with HCC.

## Introduction

Liver cancer ranks as the sixth most frequently diagnosed malignancy and the third leading cause of cancer-associated deaths worldwide, contributing 4.7% of newly diagnosed cancer cases and 8.2% of deaths annually (Bray et al., 2018). Hepatocellular carcinoma represents the most predominant pathological type of primary liver malignancy, accounting for approximately 75–85% of primary liver cancer cases (Bray et al., 2018). Surgical resection, liver transplantation, and hepatic transarterial chemoembolization (TACE) remain the mainstay curative strategies for early stage HCC patients (Knudsen et al., 2014; Fitzmorris et al., 2015). However, the majority of patients with HCC are frequently diagnosed at an advanced stage, and these treatments failed to demonstrate a survival benefit or prolong the patients’ 5-year survival rates (Galun et al., 2017; Li et al., 2019). Although the tyrosine kinase inhibitors (TKIs), sorafenib, regorafenib, and lenvatinib have been recently approved by the Food and Drug Administration (FDA) as first-line or second-line therapies for advanced HCC, their clinical efficacy remains limited (Llovet et al., 2008; Bruix et al., 2017; Kudo et al., 2018; Pinter and Peck-Radosavljevic, 2018). Thus, new personalized strategies are urgently needed for the effective treatment of advanced stage HCC patients.

Approximately 70–90% of all HCCs are associated with known underlying etiology of cirrhosis, which is predominantly attributed to chronic hepatitis B virus infection, hepatitis C virus infection, alcohol consumption, non-alcoholic steatohepatitis, and metabolic diseases (Tovoli et al., 2019). Indeed, HCC carcinogenesis independently of etiology is characterized by an inflammatory milieu and close interactions with the immune system at all stages of disease progression. Thus, the recently introduced immunotherapy has emerged as a promising therapeutic modality for the treatment of advanced HCC (Okusaka and Ikeda, 2018; Waidmann, 2018). A rapidly evolving cancer immunotherapy such as CAR-T was recognized as the first major breakthrough by Science magazine in 2013. Subsequently, in 2017, two CAR-T cell therapeutic products, Kymriah and Yescarta, were approved by the FDA as the preferred therapeutic modality for the treatment of relapsed or refractory acute lymphoblastic leukemia and refractory large B-cell lymphoma, respectively (Liu et al., 2017; Vormittag et al., 2018). CAR-T cells precisely mediate MHC-unrestricted tumor cell killing, with advantages unmatched to conventional therapies (Lim and June, 2017; June et al., 2018). Although CAR-T therapy has made breakthroughs in hematological malignancies, their therapeutic potential in solid tumors is still under pre-clinical investigation due to the scarcity of tumor-specific targets.

CD147, a transmembrane glycoprotein belonging to the immunoglobulin superfamily, is highly expressed in different cancer types, including non-small cell lung cancer, breast cancer, and HCC (Li et al., 2009). Previously, we have demonstrated that CD147 promoted the proliferation, invasion, and metastasis of HCC cells and has the potential to be used as an important prognostic and therapeutic biomarker for tumors (Wu et al., 2016; Lu et al., 2018). Moreover, a radioimmunoconjugate Iodine[^131^I]-metuximab targeting the CD147 has been developed by our team for the effective treatment of HCC; it also exhibited a significantly prolonged median time to tumor recurrence (Bian et al., 2014). Considering these findings, we envisaged that CD147 may serve as an effective target surface antigen for CAR-T immunotherapy for HCC.

However, CD147 is also considered as a tumor-associated antigen (TAA), which has the possibility to express in low quantities on non-tumor cells. CAR-T cells targeting-TAA may occur the “on-target, off-tumor” effects that cause injury of non-tumor tissues. Moreover, several dangerous side effects associated with CAR-T therapies, such as cytokine release syndrome, remain a major concern in its clinical application. So, it is necessary to tightly control the expression of CAR to minimize these side effects. Tet-On 3G is the third generation of tetracycline-inducible gene expression systems, which could reversibly turn on or off the gene expression using doxycycline (Dox). We believe the Tet-On 3G system might be an ideal choice to regulate the expression of CD147CAR both *in vitro* and *in vivo*.

Therefore, this study was initiated to investigate the anti-tumor potential of CAR-T cells targeting the CD147 using the Tet-On inducible gene system in HCC. Herein, we constructed a lentiviral vector containing the Tet-On 3G inducible system and CD147CAR domain. Then, Tet-CD147CART cells were generated through infection with lentivirus. Subsequently, the optimal dose and dynamics of Dox-regulated CAR expression on T cells were determined. Furthermore, both *in vitro* and *in vivo* therapeutic effects of Tet-CD147CART cells in HCC were evaluated.

## Materials and Methods

### Ethics Statement

The study protocols were approved by the Institutional Ethics Review Board of the Fourth Military Medical University.

### Construction of Lentiviral Vector

The single-chain variable fragment targeting CD147 (CD147-scFv) was constructed based on the sequences of humanized monoclonal antibody against CD147. The heavy-chain and light-chain variable region were connected with G_4_S linker. CD147-scFv was then fused to a human CD8 hinge, a 4-1BB cytoplasmic domain, and a CD3ζ signaling domain to constitute CD147CAR, which was under the control of Tet response element (TRE3G) promoter. An enhanced green fluorescent protein (EGFP) and CD147CAR were coexpressed at equimolar levels from a single transcript by inserting the self-cleaving P2A peptide. The Tet-On 3G system was controlled by the immediate-early cytomegalovirus (CMV) promoter, which was inserted upstream of CD147CAR in reverse orientation. Fragments were ligated using the In-Fusion cloning system (TaKaRa Bio, Shiga, Japan).

### Cell Lines

The human HCC cell line HepG2 was acquired from the American Type Culture Collection (Manassas, VA, United States). The human HCC cell line Huh-7 was obtained from the Japanese Collection of Research Bioresources (JCRB, Osaka, Japan). All cell lines were cultured in RPMI 1640 medium supplemented with 10% fetal bovine serum and 100 μg/mL of penicillin-streptomycin at 37°C in a humidified incubator with 5% CO_2_. For the preparation of HepG2-shCD147 knockdown clones, HepG2 cells were transfected with LV-shCD147 lentivirus cloned against CD147. Huh-7 cells overexpressing CD147 (Huh7-CD147) were generated by transfection with a lentivirus encoding CD147.

### Generation and Expansion of Tet-CD147CART Cells

Peripheral blood mononuclear cells were isolated from freshly donated blood of healthy donors using Ficoll-Paque by density gradient centrifugation. PBMCs were then cultured in RPMI 1640 medium containing 10% fetal bovine serum, 100 μg/mL penicillin-streptomycin, 300 IU/mL recombinant human IL-2, and 50 ng/mL OKT-3 at 37°C in a humidified incubator with 5% CO_2_. After 24 h, PBMCs were infected with encoding lentivirus and then expanded in RPMI 1640 medium in the absence of OKT-3. On the 6th day post-activation, Dox was added to the medium to a final concentration of 1000 ng/mL. The CD147CAR positive cells were detected by flow cytometry on day 7 and were used for subsequent experiments. (Dox+) Tet-CD147CART cells indicated Tet-CD147CAR-transduced PBMCs in the presence of Dox, and (Dox−) Tet-CD147CART cells indicated Tet-CD147CAR-transduced PBMCs in the absence of Dox.

### Dynamic of Tet-CD147CAR Expression

For dose-dependent curve of Tet-CD147CAR expression, different concentrations of Dox were added to the medium on the 6th day after T cell activation. The mean fluorescence intensity (MFI) of Tet-CD147CART cells was determined using flow cytometry after 24 h. For time-dependent curve of Dox-induced Tet-CD147CAR expression, 1000 ng/mL of Dox was added to the medium on the 6th day after T cell activation. The MFI of Tet-CD147CART cells was determined using flow cytometry after 0, 4, 8, 12, 24, 32, and 48 h, respectively. For time-dependent curve of Tet-CD147CAR expression after Dox elimination, 1000 ng/mL of Dox was added to the medium for 24 h on the 6th day after T cell activation. Subsequently, Dox was eliminated and the MFI of Tet-CD147CART cells was determined using flow cytometry after 0, 12, 24, 48, 72, and 96 h.

### Cell Proliferation Assay

For cytokine-dependent cell proliferation, 5 × 10^5^ (Dox+) Tet-CD147CART cells, (Dox−) Tet-CD147CART cells, and PBMCs were cultured in RPMI 1640 medium supplemented with 300 IU/mL IL-2. Growth was assessed by cell counts every 24 or 48 h, and the proliferation fold was calculated. For antigen-dependent cell proliferation, 2 × 10^5^ (Dox+) Tet-CD147CART cells, (Dox−) Tet-CD147CART cells, and PBMCs were co-cultured with 4 × 10^4^ Huh7-CD147 cells in RPMI 1640 medium supplemented with 100 IU/mL IL-2. Growth was assessed every 24 or 48 h, and proliferation fold was calculated.

### Lactate Dehydrogenase Release Assay

Lactate dehydrogenase release assay was performed to measure the cytotoxicity of (Dox+) Tet-CD147CART cells, (Dox−) Tet-CD147CART cells, and PBMCs against tumor cells. Effector (E) and tumor (T) cells were co-cultured for 16 h in 96-well plates at indicated E:T ratios. Then, the supernatant was harvested after centrifugation, and the release level of LDH was measured using the Cytotoxicity Detection Kit^PLUS^ (LDH) (Promega, Madison, WI, United States) according to the manufacturer’s protocol.

### Cytokine Secretion Assay

Cytokine secretion by (Dox+)Tet-CD147CART cells, (Dox−) Tet-CD147CART cells, and PBMCs against tumor cells was analyzed with flow cytometry. Effector cells and tumor cells were co-cultured for 16 h in 96-well plates at a ratio of 10:1. Then, the supernatant was harvested after centrifugation, and the secretion levels of IFN-γ, TNF-α, IL-2, IL-4, IL-6, and IL-17A were measured using AimPlex^TM^ Analyte Kit (QuantoBio, Beijing, China) according to the manufacturer’s instruction.

### Xenograft Model of a CD147-Positive Tumor

Six- to eight-week-old nude mice were procured from Vitalstar Biotechnology (Beijing, China) and bred and kept in a specialized pathogen-free facility with daily monitoring. The animals were cared for in accordance with the guide for the care and use of laboratory animals. All procedures and animal experiments were approved by the Animal Care and Use Committee of the Fourth Military Medical University. A xenograft model was established through subcutaneous injection of 5 × 10^6^ Huh-7 cells. Animals were monitored daily, and tumor size was measured once a week. About 2 weeks post-inoculation, tumor-bearing mice with a tumor size of approximately 5 mm were randomized into (Dox+) group, (Dox−) group, and PBMC group (*n* = 6 for each group). To the (Dox+), (Dox−), and PBMC groups, 5 × 10^6^ (Dox+) Tet-CD147CART cells, (Dox−) Tet-CD147CART cells, and PBMCs were administered intratumorally for the first time, respectively. Subsequently, the three kinds of effector cells were injected intratumorally every 3 days for a total of four times. Moreover, 3 days before the first CAR-T cell reinfusion, 2 mg/mL Dox was added to the drinking water of nude mice in the (Dox+) group.

### H&E and Immunohistochemistry

Mice were sacrificed and tumor tissues were fixed with 10% formalin followed by embedding in paraffin. Tissue sections (4 μm, thick) were sectioned from the paraffin blocks, stained with hematoxylin and eosin (H&E) or immunohistochemistry. In particular, immunohistochemistry was performed by staining with anti-GFP antibody (sc-9996, Santa Cruz Biotechnology, Dallas, TX, United States) or anti-human CD8 antibody (85336, Cell Signaling Technologies, Danvers, MA, United States) using a streptavidin-peroxidase staining kit (Zhongshan Jinqiao, Co., Beijing, China).

### FACS Analysis

All samples were analyzed using flow cytometry with the FACS Aria (BD Biosciences, San Jose, CA, United States) instrument, and data were analyzed using FlowJo software (TreeStar, Ashland, OR, United States).

### Statistical Analysis

Statistical analyses were performed using GraphPad Prism v7.0 software (GraphPad Software, La Jolla, CA, United States) and SPSS 23.0 (SPSS, Inc., Chicago, IL, United States). Quantitative data are presented as the mean ± standard error (SE) of at least three independent experiments. For *in vitro* and *in vivo* comparisons, data were analyzed using unpaired *t*-tests. *P*-value < 0.05 was considered statistically significant.

## Results

### Design and Construction of the Tet-CD147CAR Lentiviral Vector

**FIGURE 1:**
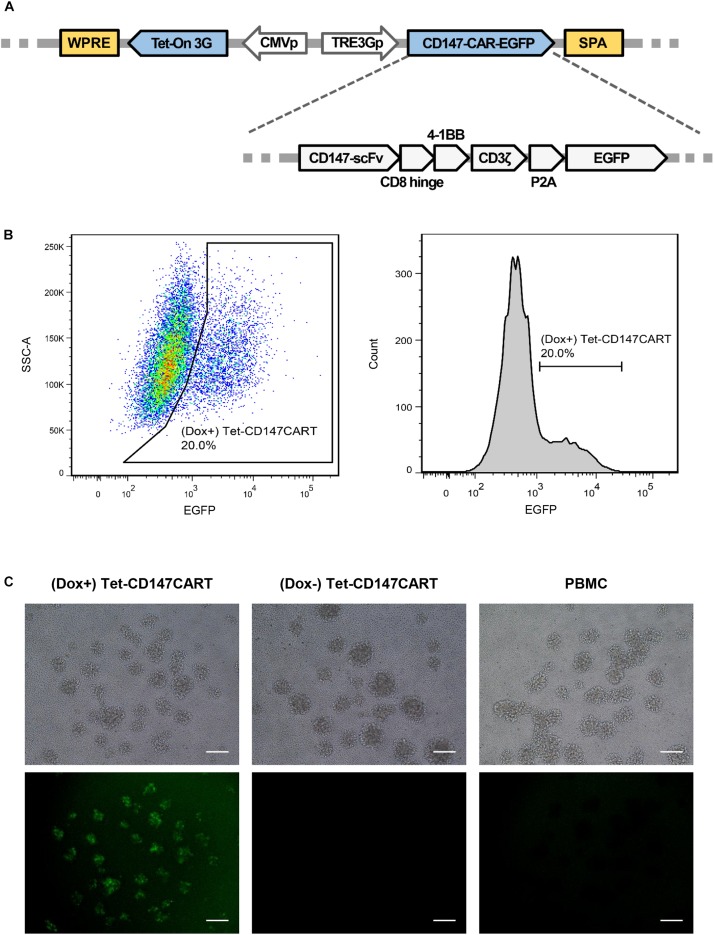
Construction of the Tet-CD147CAR lentiviral vector and the expression of Tet-CD147CART cells in the presence of Dox. **(A)** Schematic outline of the Tet-CD147CAR lentiviral vector. **(B,C)** PBMCs were infected by Tet-CD147CAR lentivirus. Tet-CD147CAR-positive expression on T cells in the presence of Dox was determined by FACS analysis **(B)** and fluorescence microscopy **(C)**. Scale bars: 500 μm.

To develop inducible CAR-T cells targeting CD147, a lentiviral vector was constructed. The CAR fragment was generated and designated as CD147-CAR-EGFP, which comprised CD147-scFv, human CD8 hinge domain, 4-1BB cytoplasmic domain, CD3ζ signaling domain, P2A peptide, and EGFP. Tet-On 3G and TRE3G were amplified from pCMV-Tet3G and pTRE3G, respectively, and then inserted into the vector using the In-Fusion cloning system (Figure 1A). The construction was confirmed by sequencing. Subsequently, the Dox inducible CD147CAR encoding lentivirus was generated and named as LV-Tet-CD147CAR.

### Construction of Tet-CD147CART Cells

To generate the Tet-CD147CART cells, PBMCs were isolated from healthy donors. In order to improve the transfection efficacy, PBMCs were activated by OKT-3, an antibody targeting CD3, for 24 h. FACS analysis showed that CD25 and CD69 were significantly increased after OKT-3 activation (Supplementary Figure 1). Further, using FACS, the transfection efficiency of Tet-CD147CART cells after lentiviral transfection was examined. The results showed that the expression of Tet-CD147CAR on T cells could be induced effectively after the addition of Dox in the culture medium, and the transfection efficiency ranged from 10 to 20% due to individual differences (Figure 1B). Consistently, fluorescence imaging also confirmed these findings as illustrated in Figure 1C. In addition, the subpopulation analysis indicated that (Dox+) Tet-CD147CART cells were mainly composed of CD4 + T cells (73.6%) and CD8 + T cells (19.5%) (Supplementary Figure 2). Taken together, these results indicated that the LV-Tet-CD147CAR could infect human T cells to effectively generate Tet-CD147CART cells, and the Dox could significantly induce the expression of CD147CAR in transfected T cells.

### Dynamics of Dox-Induced CD147CAR Expression

**FIGURE 2:**
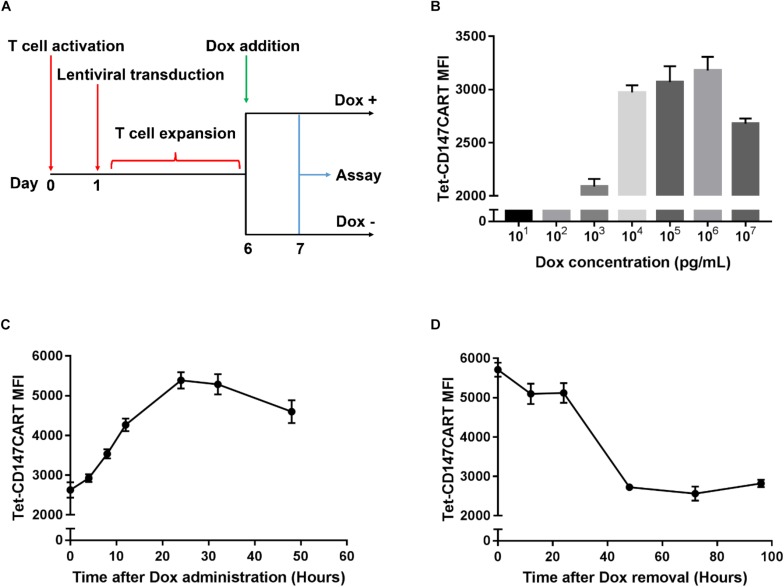
Dynamic of Tet-CD147CAR expression upon Dox administration or elimination. **(A)** Schematic representation of the construction and assay of Tet-CD147CART cells in the presence or absence of Dox. **(B)** Dose-dependent curve of Tet-CD147CAR expression after the addition of Dox at 24 h. **(C)** Time-dependent curve of Dox-induced Tet-CD147CAR expression after the addition of 1000 ng/mL Dox. **(D)** Time-dependent curve of Tet-CD147CAR expression after Dox elimination.

To determine whether Dox-induced CD147CAR expression was in a dose-dependent manner, Tet-CD147CART cells were supplemented with different concentrations of Dox in the culture medium (Figure 2A). As illustrated in Figure 2B, the MFI of Tet-CD147CART cells reached a peak at 1000 ng/mL of Dox concentration and were considered appropriate to obtain maximal CAR expression; therefore, this concentration was selected as the optimum concentration for subsequent experiments. Besides, the time-dependent relationship of Dox-induced CD147CAR expression was detected by FACS analysis. The results indicated that CAR expression peaked at 24 h after administration of Dox and returned to baseline level at 48 h after removal of Dox from the culture medium (Figures 2C,D).

### Expansion of Tet-CD147CART Cells

**FIGURE 3:**
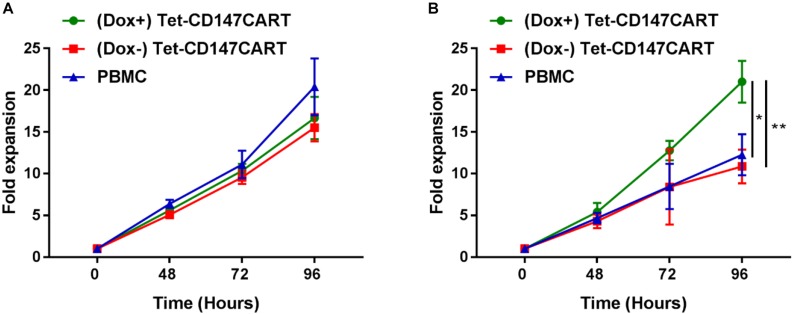
Proliferation of (Dox+) Tet-CD147CART cells, (Dox−) Tet-CD147CART cells, and PBMCs *in vitro*. **(A)** Cytokine-dependent cell proliferation. **(B)** Antigen-dependent cell proliferation. *n* = 3, ^∗^*P* < 0.05, ^∗∗^*P* < 0.01.

To investigate the expansion ability of Tet-CD147CART cells *in vitro*, the cell proliferation was measured by counting viable cells. No significant differences in the proliferation rates of (Dox+) Tet-CD147CART cells, (Dox−) Tet-CD147CART cells, or PBMCs were observed within 96 h of cell culture (Figure 3A). These results indicated that the expression of CD147CAR did not influence the proliferative efficacy of lentivirus-transduced cells.

Furthermore, we also examined the antigen-dependent proliferative capacity of Tet-CD147CART cells with or without Dox. Tet-CD147CART cells were co-cultured with Huh7-CD147 cells at a 5:1 ratio with IL-2, and the viability of CAR-T cells and PMBCs was also measured by counting the viable cells. As shown in Figure 3B, the expansion of (Dox+) Tet-CD147CART cells was significantly increased compared to that of (Dox−) Tet-CD147CART cells (*P* < 0.01) and PBMCs (*P* < 0.05).

### CD147 Dependent Cytotoxicity of Tet-CD147CART Cells

**FIGURE 4:**
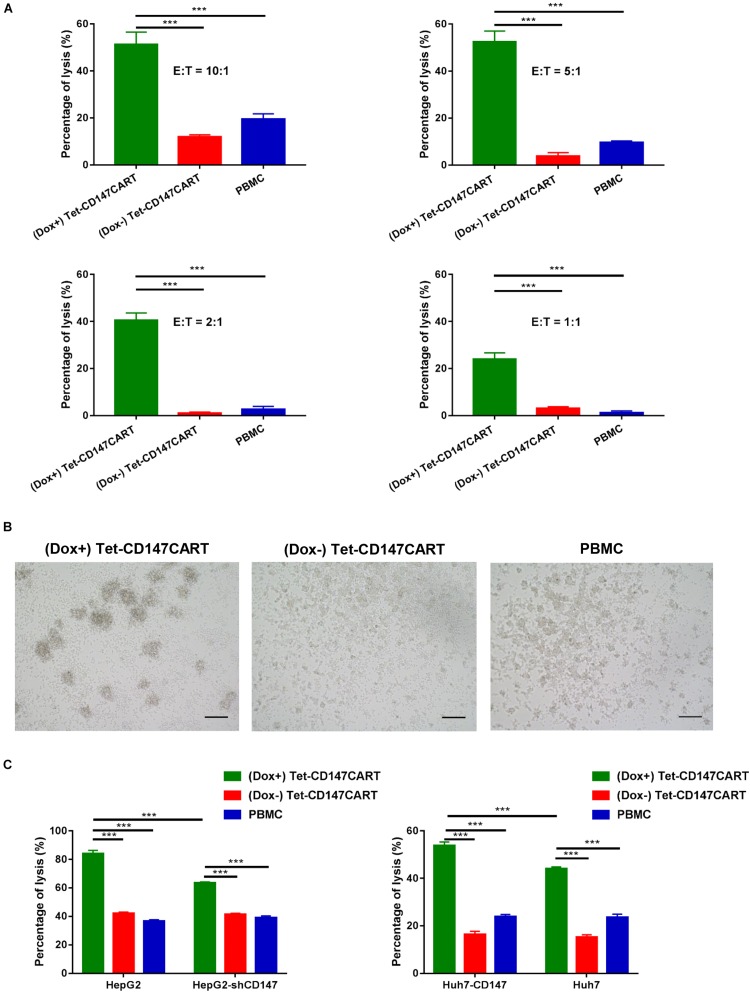
Cytotoxicity of (Dox+) Tet-CD147CART cells, (Dox−) Tet-CD147CART cells, and PBMCs *in vitro*. **(A)** Percentages of HepG2 cells lysis when co-cultured with (Dox+) Tet-CD147CART cells, (Dox−) Tet-CD147CART cells, and PBMCs at different E:T ratios were determined using lactate dehydrogenase release assay. *n* = 4, ^∗∗∗^*P* < 0.001. **(B)** Morphological changes of HepG2 cells when co-cultured with three kinds of effector cells at an E:T ratio of 10:1 as confirmed with microscopy. Scale bars: 500 μm. **(C)** Antigen-dependent cytotoxicity of CAR-T cells. Comparisons of the percentage of HepG2 and HepG2-shCD147 cells lysis (left) or Huh7-CD147 and Huh7 cells lysis (right) when co-cultured with three kinds of effector cells at an E:T ratio of 10:1 were determined using LDH release assay. *n* = 3, ^∗∗∗^*P* < 0.001.

In order to effectively characterize the activity of Tet-CD147CART cells, the optimal effector-target ratio was first determined through *in vitro* cytotoxicity experiments. The cytotoxicity of Tet-CD147CART cells in the presence of Dox was determined using LDH-release assay at different E:T ratios of 10:1, 5:1, 2:1, and 1:1. CD147-postive HepG2 was chosen as a target cell line. The results presented in Figure 4A indicate that the cytolytic activity of three kinds of effector cells against HepG2 cells gradually increased with the increase in E:T ratio. Particularly, compared with (Dox−) Tet-CD147CART cells and PBMCs, (Dox+) Tet-CD147CART cells exhibited noticeably higher cytotoxicity at all E:T ratios. Morphological data also confirmed that the percentage lysis of HepG2 cells increased markedly when co-cultured with (Dox+) Tet-CD147CART cells compared to (Dox−) Tet-CD147CART cells and PBMCs, respectively (Figure 4B).

To further explore the cytotoxic ability of Tet-CD147CART cells, we established two stable cell lines, HepG2-shCD147 and Huh7-CD147, with CD147 knocked down and overexpressed, respectively (Supplementary Figures 3A,B). For (Dox−) Tet-CD147CART cells and PBMCs, no significant differences were observed when they co-cultured with HepG2 or HepG2-shCD147 cells. However, (Dox+) Tet-CD147CART cells exhibited significantly lower cytotoxicity when co-cultured with HepG2-shCD147 cells (Figure 4C). Similar findings were observed with Huh-7 and Huh7-CD147 cells, and only the cytotoxicity of (Dox+) Tet-CD147CART cells was positively correlated with the expression level of CD147 (Figure 4C). Taken together, these results demonstrated that Tet-CD147CART cells exhibit significant cytotoxicity against HCC cells in the presence of Dox in an antigen-dependent manner.

### Cytokines Secretion by Tet-CD147CART Cells

**FIGURE 5:**
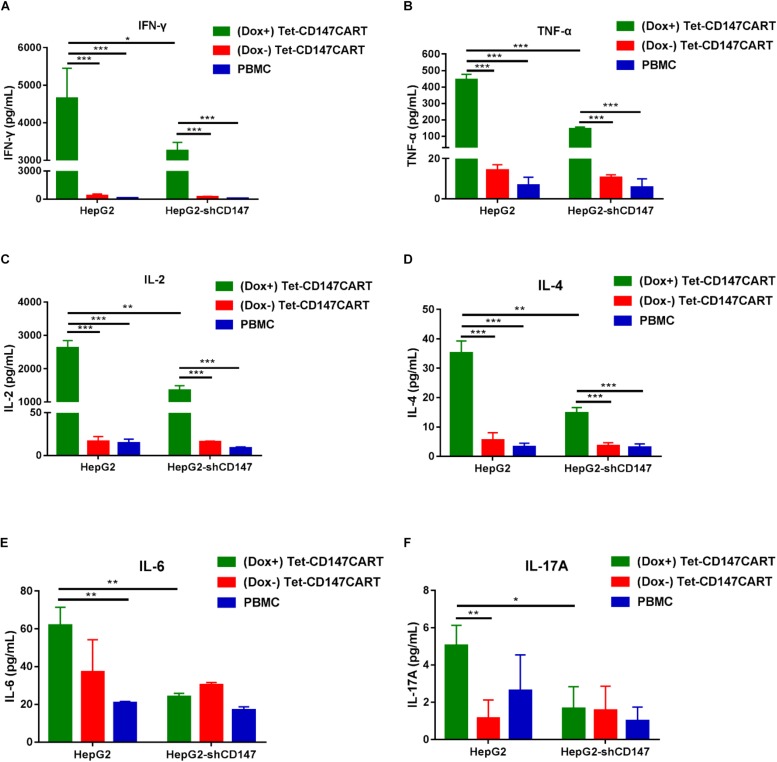
Cytokine secretion by (Dox+) Tet-CD147CART cells, (Dox−) Tet-CD147CART cells, and PBMCs *in vitro*. **(A–F)** After being co-cultured with HepG2 and HepG2-shCD147 cells at an E:T ratio of 10:1 for 16 h, cytokine secretion levels of IFN-γ, TNF-α, IL-2, IL-4, IL-6, and IL-17A from effector cells in the supernatants were determined by flow cytometry. *n* = 3, ^∗^*P* < 0.05, ^∗∗^*P* < 0.01, ^∗∗∗^*P* < 0.001.

Cytokine secreted by CAR-T cells plays a critical role in the CAR-T cell therapy; it not only enhances anti-tumor efficacy but is also recognized to elicit severe adverse effects. To determine the cytokine secretion by Tet-CD147CART cells, six cytokines were measured after effector cells recognized tumor cells. As shown in Figure 5, different cytokines including IFN-γ, TNF-α, IL-2, and IL-4 were secreted by Tet-CD147CART cells and were significantly increased in the presence of Dox when co-cultured with HepG2 cells. Interestingly, when the expression of CD147 was knocked down in HepG2 cells, the cytokine release level of (Dox+) Tet-CD147CART cells was also reduced remarkably, while the cytokine release level of (Dox−) Tet-CD147CART cells and PBMCs showed no significant differences. Moreover, (Dox+) Tet-CD147CART cells co-cultured with Huh-7 cells exhibited higher secretion levels of different cytokines including IFN-γ, IL-2, IL-4, IL-6, and IL-17A compared to (Dox−) Tet-CD147CART cells and PBMCs (Supplementary Figure 4). These results indicated that Dox can induce the expression and function of CD147CAR on Tet-CD147CART cells, which can release various immunostimulatory cytokines in an antigen-dependent manner to enhance the anti-tumor effect.

### Anti-tumor Effect of Tet-CD147CART Cells *in vivo*

**FIGURE 6:**
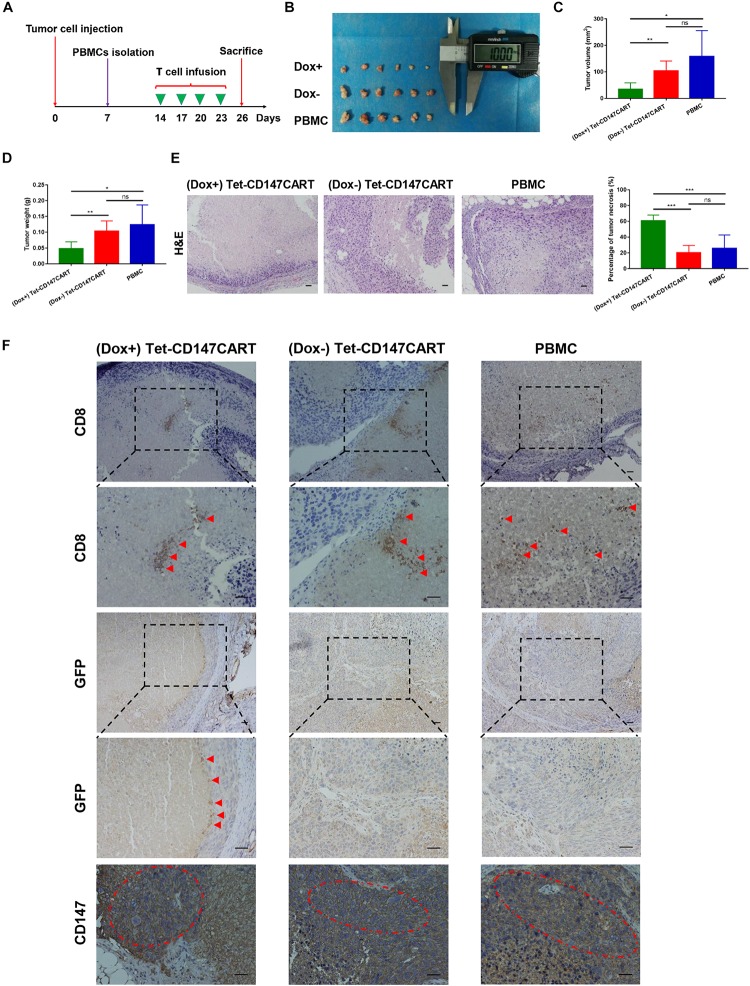
Anti-tumor effect of (Dox+) Tet-CD147CART cells, (Dox−) Tet-CD147CART cells, and PBMCs *in vivo*. **(A)** Schematic representation of the *in vivo* experimental procedure. Nude mice were inoculated with 5 × 10^6^ Huh-7 cells subcutaneously on day 0 and randomized into three groups on day 14. PBMCs were isolated from healthy donors and then activated, infected, and expanded. The 5 × 10^6^ effector cells were injected intratumorally four times on days 14, 17, 20, and 23. Subsequently, nude mice were sacrificed on day 26. **(B)** Tumor tissues obtained from nude mice on day 26. Dox+ = (Dox+) Tet-CD147CART cells, Dox− = (Dox−) Tet-CD147CART cells. **(C–E)** Tumor volume, tumor weight, and necrotic area in each group. *n* = 6, ^∗^*P* < 0.05, ^∗∗^*P* < 0.01, ^∗∗∗^*P* < 0.001, ns = not statistically significant. **(F)** The distribution of human CD8 + T cells and Tet-CD147CART cells and CD147 expression were detected in tumor tissues with immunohistochemistry staining. The red arrows indicate CD8 + T cells or Tet-CD147CART cells. The red dotted ellipses indicate positive expression of CD147 in tumor tissues. Scale bars: 100 μm.

To further assess the anti-tumor effect of Tet-CD147CART cells *in vivo*, we used a mouse model of nude mice bearing subcutaneous Huh-7 cells xenograft. Two weeks post-inoculation, mice in the (Dox+), (Dox−), and PBMC groups were treated with (Dox+) Tet-CD147CART cells, (Dox−) Tet-CD147CART cells, and PBMCs, respectively. All mice were administered intratumorally every 3 days for four times (Figure 6A). At 26 days post-inoculation, all mice were sacrificed and tumors were collected as shown in Figure 6B. Tumor volume and weight were measured, and the results indicated that tumors in the mice which received (Dox+) Tet-CD147CART cells were significantly reduced compared with those of mice that received (Dox−) Tet-CD147CART cells or PBMCs. However, no significant difference was observed in the tumor growth between the latter two groups (Figures 6C,D). H&E staining analysis showed that Tet-CD147CART cell treatment with Dox exhibited significantly higher necrosis in tumor tissue compared with the other two groups as observed from the consecutive tissue sections (Figure 6E). Using anti-human CD8 mAb, anti-GFP mAb, and anti-CD147 mAb, we examined the distribution of human CD8 + T cells and Tet-CD147CART cells and CD147 expression in tumor tissues. As illustrated in Figure 6F, human CD8 + T cells were observed in necrotic tissues of tumors and CD147 was positive in all the three groups, while the Tet-CD147CART cells were only detected in the (Dox+) group, and we believe these Tet-CD147CART cells caused the enhanced tumor necrosis in (Dox+) group. All in all, these results indicated that Tet-CD147CART cells could effectively kill cancer cells in the HCC xenograft model, and the function of Tet-CD147CART cells could be regulated by Dox administration both *in vitro* and *in vivo*.

## Discussion

Conceivably, enlightened with the unprecedented therapeutic efficacy of CAR-T cell therapy in treating malignant hematological diseases, CAR-T cell immunotherapy finds its utility in solid tumors—however, with elusive efficacy (Lee et al., 2015; Filley et al., 2018; Neelapu et al., 2018). Particularly, the lack of specific antigen targets remains the major obstacle of CAR-T-based immunotherapy in the targeting of solid tumors (Li and Zhao, 2017). Presently, multiple TAAs including GPC3, MUC1, AFP, and EpCAM are under investigation for HCC (Jiang et al., 2016; Zhang et al., 2019). In the present study, we for the first time report the anti-tumor effect of CAR-T cells targeting CD147 in HCC. CD147 is highly expressed in HCC with a positive rate of 75–80% (Chen et al., 2006; Li et al., 2009). Previously, our team has developed radioimmunoconjugate [^131^I]-labeled metuximab targeting CD147 for the treatment of HCC. Thus, CD147 may also be a potential promising antigen target for CAR-T therapy in HCC.

However, since CD147 is a TAA, the high degree of upregulation of CD147 allows for preferential targeting of tumor cells, there is still the possibility that CD147-CAR recognizes the CD147-expressed normal tissues. This on-target/off-tumor effect may cause severe toxicity of CAR-T cell therapy and limit the therapeutic effects of CAR-T cell for solid tumors. Regional administration, which could increase the residency of CAR-T cells in tumor tissue and decrease the systemic absorption, may be a feasible and superior strategy to prevent non-specific destruction of normal tissues and reduce the risk of off-target toxicities (Parente-Pereira et al., 2011; Jiang et al., 2016; Li and Zhao, 2017; Filley et al., 2018; Neelapu et al., 2018; Zhang et al., 2019). Nowadays, nearly half of CAR-T clinical trials for HCC use regional administration, such as hepatic artery infusion or ultrasound-guided intra-tumor injection. However, regional administration is not suitable for deep-seated tumors or metastatic diseases that have multiple lesions in multiple sites, and thus, its use is greatly limited in clinical applications. So, other strategies, including dual-targeting CAR and affinity-tuned CAR, were developed to avoid the on-target/off-tumor toxicity. Dual-targeting CAR-T could eliminate tumor cells that express two antigens but did not affect normal tissue expressing either antigen alone (Kloss et al., 2013). And affinity-tuned CAR lowers the affinity of CAR to antigen, which renders CAR-T cells to distinguish tumor from normal cells (Caruso et al., 2015; Liu et al., 2015; Arcangeli et al., 2017). However, both dual-targeting CAR and affinity-tuned CAR were highly dependent on the type and density of antigen expressed on tumor cells, which will influence the therapeutic effects and off-tumor toxicity greatly. To control the off-tumor toxicity of CAR-T cell therapeutic, several suicide genes, including caspase-9 and HSV-tk, were introduced to eliminate uncontrolled activation CAR-T cells. In a recent study, Gargett and Brown (2014) constructed an inducible caspase-9 safety switch which could induce the apoptosis of CAR-T in the presence of drug AP1903, and the novel CAR-T cell was investigated in a phase I trial in solid tumor patients (Yu et al., 2019).

Here, to better control the expression and function of CD147CAR on T cells and minimize the adverse events, we first integrated the Tet-On inducible gene system with the CAR structure in HCC. Compared with other strategies, CD147CAR expression can be effectively, flexibly, and sensitively regulated by the Tet-On 3G system (Loew et al., 2010; Chen et al., 2017; Hoseini and Cheung, 2017). Once an intolerable adverse reaction occurs, the supply of Dox can be immediately terminated and the expression of CD147CAR on T cells will return to baseline within 24–48 h. Therefore, Tet-CD147CART cells not only exhibit efficient anti-tumor effects but also showed greater safety than conventional CAR-T cells.

The infiltration and residency of CAR-T cells into tumor masses play a crucial anti-tumor role in solid tumors (Sackstein et al., 2017). In previous research, infiltration caused by CAR-T cells into tumor tissue represented a major challenge in the treatment of solid tumors. Predominantly, HCC occurs in patients with underlying chronic liver disease and cirrhosis; this physical barrier and immunosuppressive milieu of the liver makes CAR-T cells less likely to infiltrate into the tumor (Pardee and Butterfield, 2012; Makarova-Rusher et al., 2015). Conceivably, intratumoral administration as the reinfusion method was chosen in the study, with which a significant anti-tumor efficacy of CAR-T cells was achieved. These results suggested that local treatment of solid tumors with CAR-T cells is a feasible strategy and can be implicated in the clinical setting for patient care. Notably, for the local treatment of HCC, CAR-T cells can be reinfused through hepatic artery cannula, thus ensuring its safety and effectiveness. Moreover, to further improve the therapeutic effect, how to transform the CAR structure to achieve better CAR-T cells homage and residency into the targeted tumor tissue is the next step of exploration. C–C chemokine receptor type 5 (CCR5) was the first chemokine receptor reported to significantly promote T-cell infiltration into tumor tissues (Uekusa et al., 2002). Since then, several correlative studies on chemokines and chemokine receptors expressed on effector cells or tumor cells have been conducted (Sharma et al., 2013; Mikucki et al., 2015; Mohs et al., 2017). Adachi et al. (2018) reported that expression of interleukin-7 (IL-7) and C–C motif chemokine ligand 9 (CCL19) on CAR-T cells significantly promoted immune cell infiltration and CAR-T cell survival in the tumor. In addition, RUNX3, a key transcription factor, can promote T-cell residency in non-lymphoid tissues, which enhanced the therapeutic effect of adoptive cell therapy targeting cancer (Milner et al., 2017). Thus, based on the structure of existing Tet-CD147CAR, super CAR-T cells that express chemokine receptors such as CCR5 and transcription factor RUNX3 in tandem are expected to demonstrate better long-term therapeutic effects against HCC.

Considering the complexity of the tumor immunosuppressive microenvironment, single CAR-T therapy still presents significant limitations to the treatment of solid tumors. Ample evidence proves that tumors can evade immune surveillance by expressing immune co-inhibitory receptors, such as programed cell death protein 1 (PD-1), cytotoxic T lymphocyte-associated protein 4 (CTLA-4), lymphocyte activation gene 3 (LAG3), and T cell immunoglobulin-3 (TIM3) (Huang et al., 2017; Xu et al., 2018). Therefore, the combination therapy of Tet-CD147CART cells and immune checkpoint blockade using monoclonal antibody or genetic approaches can be envisaged as promising strategies in solid tumors including HCC. Furthermore, Wing et al. (2018) reported that oncolytic viruses could significantly improve CAR-T cell activation and proliferation, and the combinatorial approach improved anti-tumor efficacy and prolonged survival in mouse tumor models. Collectively, these findings indicated that combination strategies of CAR-T therapy, immune checkpoint blockade, and oncolytic virus, with conventional methods such as surgery and chemotherapy, are the inevitable choice for more personalized therapy in solid tumors in the near future. Noticeably, how to accurately assess the patient’s condition and use personalized combination therapy at the right time will be a crucial challenge for immunologists and clinicians.

In summary, the Tet-On inducible gene system to regulate the expression and function of CAR on T cells in solid tumors was utilized in the study, highlighting a novel approach for the application of CAR-T cell therapy targeting TAAs. Moreover, we constructed novel CD147-CART cells for the first time and demonstrated their anti-tumor efficacy on HCC *in vitro* and *in vivo*. The safety and efficacy of intratumoral administration used in this study also provide viable evidence in support of the potential benefits and translation of this CAR-T cell targeting CD147 strategy for the more personalized clinical treatment of HCC in the future. Furthermore, how to design a super CAR-T that can improve the proliferation and infiltration ability and how to choose the most suitable combination therapy strategy still need further investigation.

## Data Availability Statement

The raw data supporting the conclusions of this manuscript will be made available by the authors, without undue reservation, to any qualified researcher.

## Ethics Statement

The study protocols were approved by the Institutional Ethics Review Board of the Fourth Military Medical University.

## Author Contributions

HB, Z-NC, and DW contributed to the study design and manuscript revision. R-YZ, DW, and Z-KL performed the experiments and wrote the manuscript. Y-LY, WW, Z-YZ, J-JL, and ZZ provided experimental technical support. All authors read and approved the final manuscript.

## Conflict of Interest

The authors declare that the research was conducted in the absence of any commercial or financial relationships that could be construed as a potential conflict of interest.

## Abbreviations

CAR-T: chimeric antigen receptor T cell
Dox: doxycycline
HCC: hepatocellular carcinoma
H&E: hematoxylin and eosin
LDH: lactate dehydrogenase
MHC: major histocompatibility complex
PBMCs: peripheral blood mononuclear cells
TRE3G: Tet response element.
