# Supplementary figures and images for: Doxycycline Inducible Chimeric Antigen Receptor T Cells Targeting CD147 for Hepatocellular Carcinoma Therapy
Source: Front Cell Dev Biol. 2019 Oct 11;7:233. doi: 10.3389/fcell.2019.00233 (PMC6798074; doi:10.3389/fcell.2019.00233)

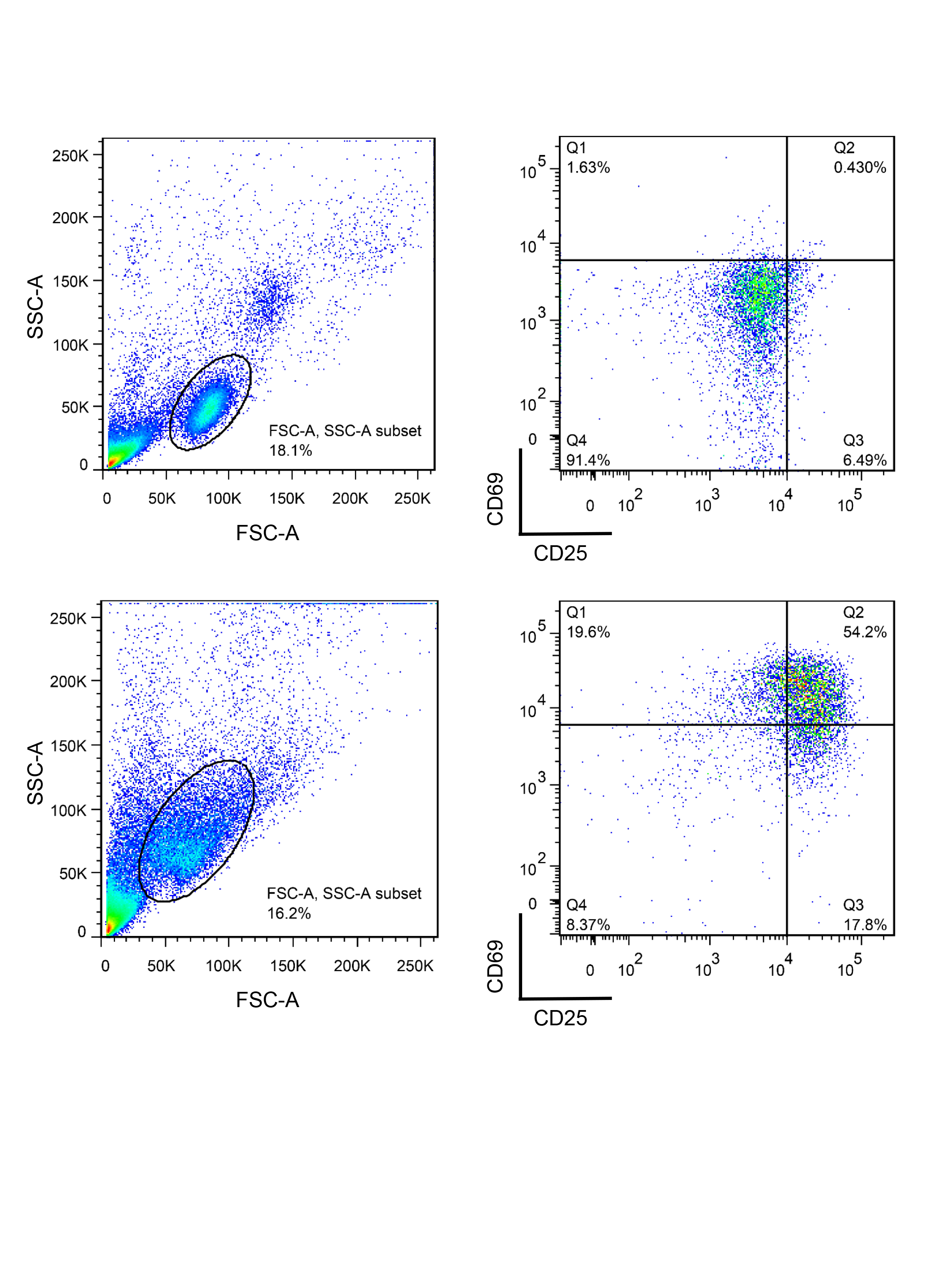

Supplement: Supplementary file 1 [file Image_1.TIF]

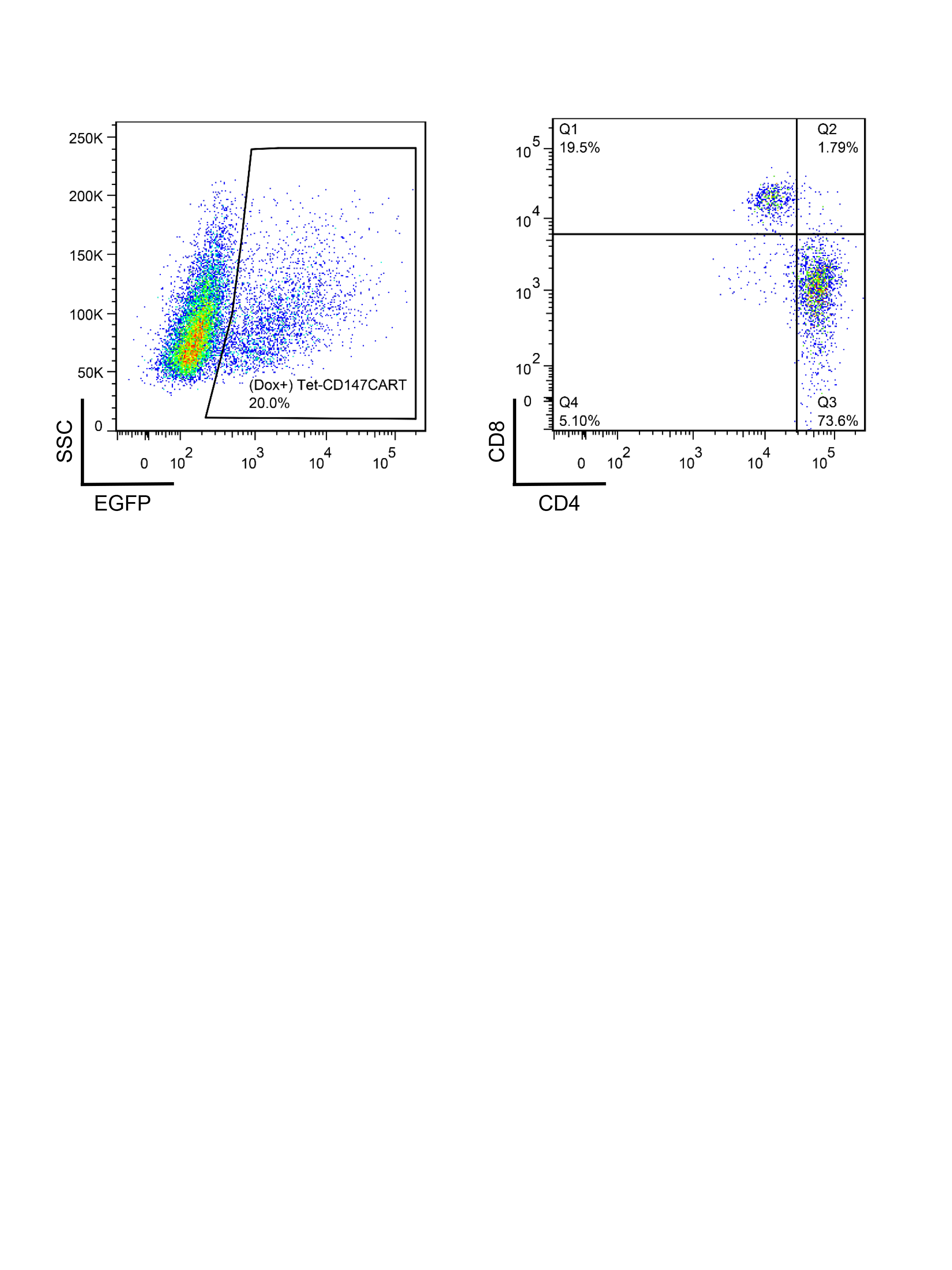

Supplement: Supplementary file 2 [file Image_2.TIF]

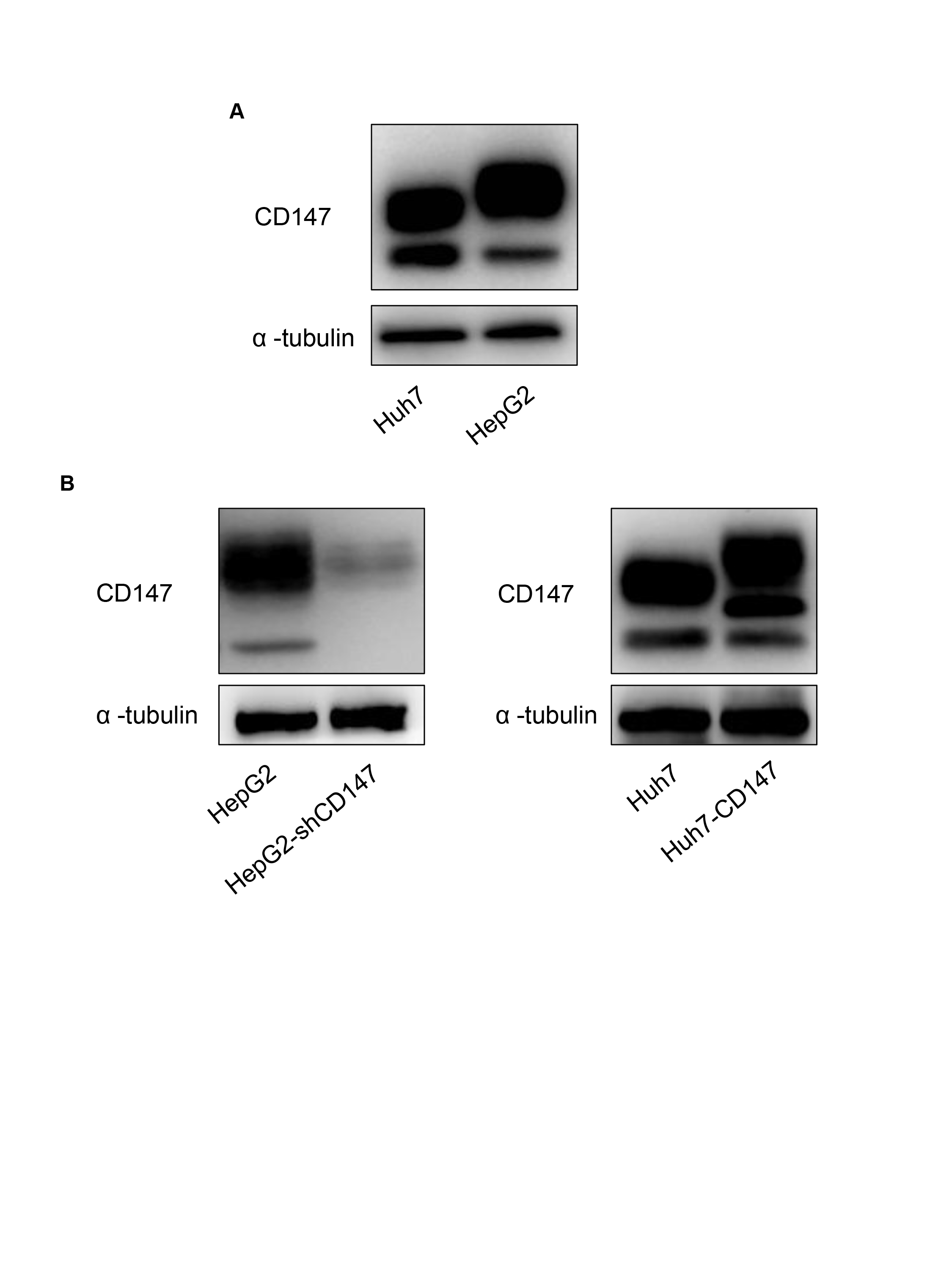

Supplement: Supplementary file 3 [file Image_3.TIF]

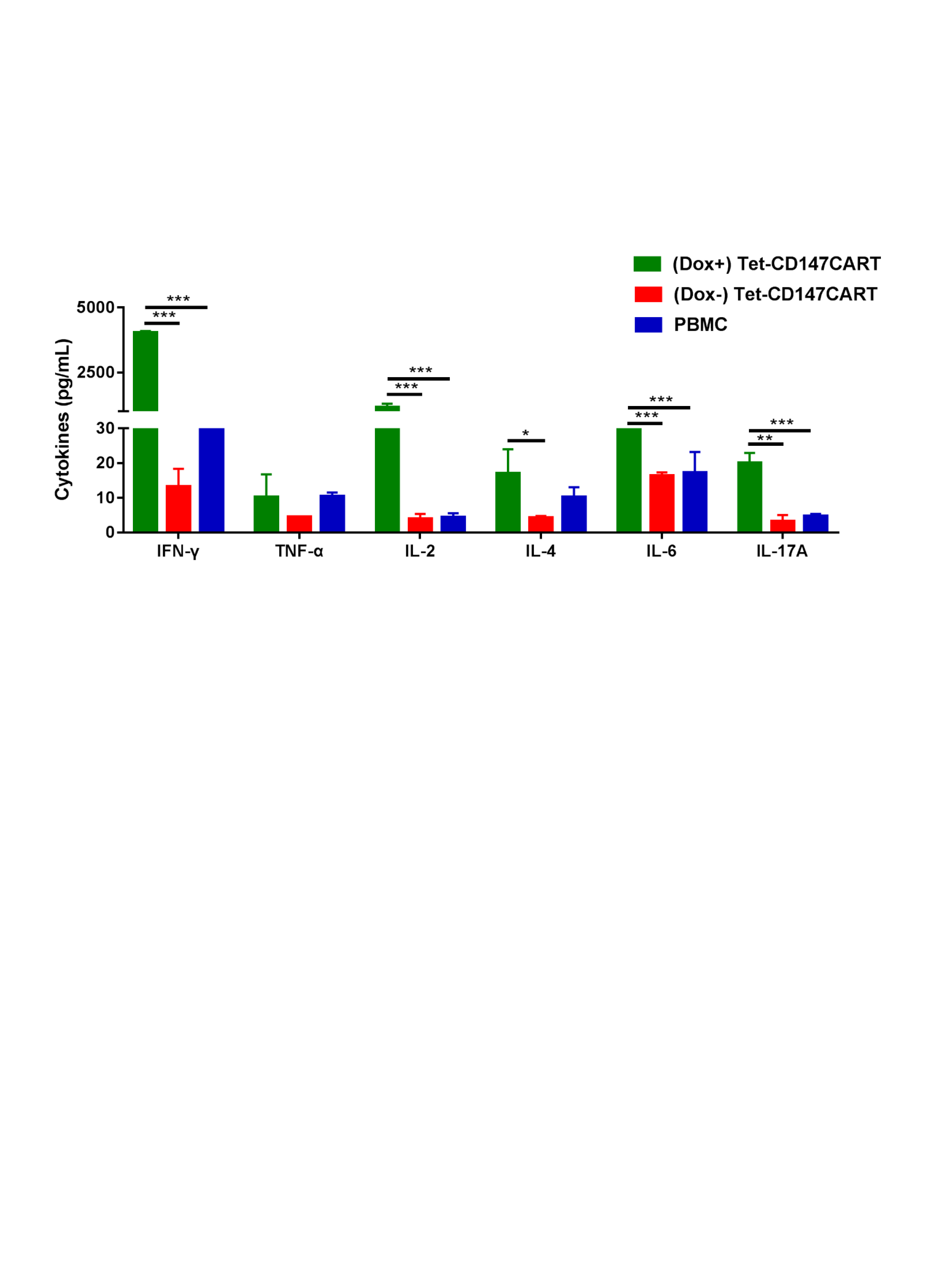

Supplement: Supplementary file 4 [file Image_4.TIF]
