# Supplementary material for: Doxycycline Inducible Chimeric Antigen Receptor T Cells Targeting CD147 for Hepatocellular Carcinoma Therapy
Source: Front Cell Dev Biol. 2019 Oct 11;7:233. doi: 10.3389/fcell.2019.00233 (PMC6798074; doi:10.3389/fcell.2019.00233)
Supplement: Supplementary file 5 [file Table_1.docx]

***Supplementary Material***

**Doxycycline Inducible Chimeric Antigen Receptor T Cells Targeting CD147 for Hepatocellular Carcinoma Therapy**

**Ren-Yu Zhang, Ding Wei*, Ze-Kun Liu, Yu-Le Yong, Wei Wei, Zhi-Yun Zhang, Jian-Jun Lv, Zhao Zhang, Zhi-Nan Chen*, Huijie Bian***

***Correspondence:** Huijie Bian: hjbian@fmmu.edu.cn; Zhi-Nan Chen: [znchen@fmmu.edu.cn](mailto:znchen@fmmu.edu.cn); Ding Wei: wdcq@163.com


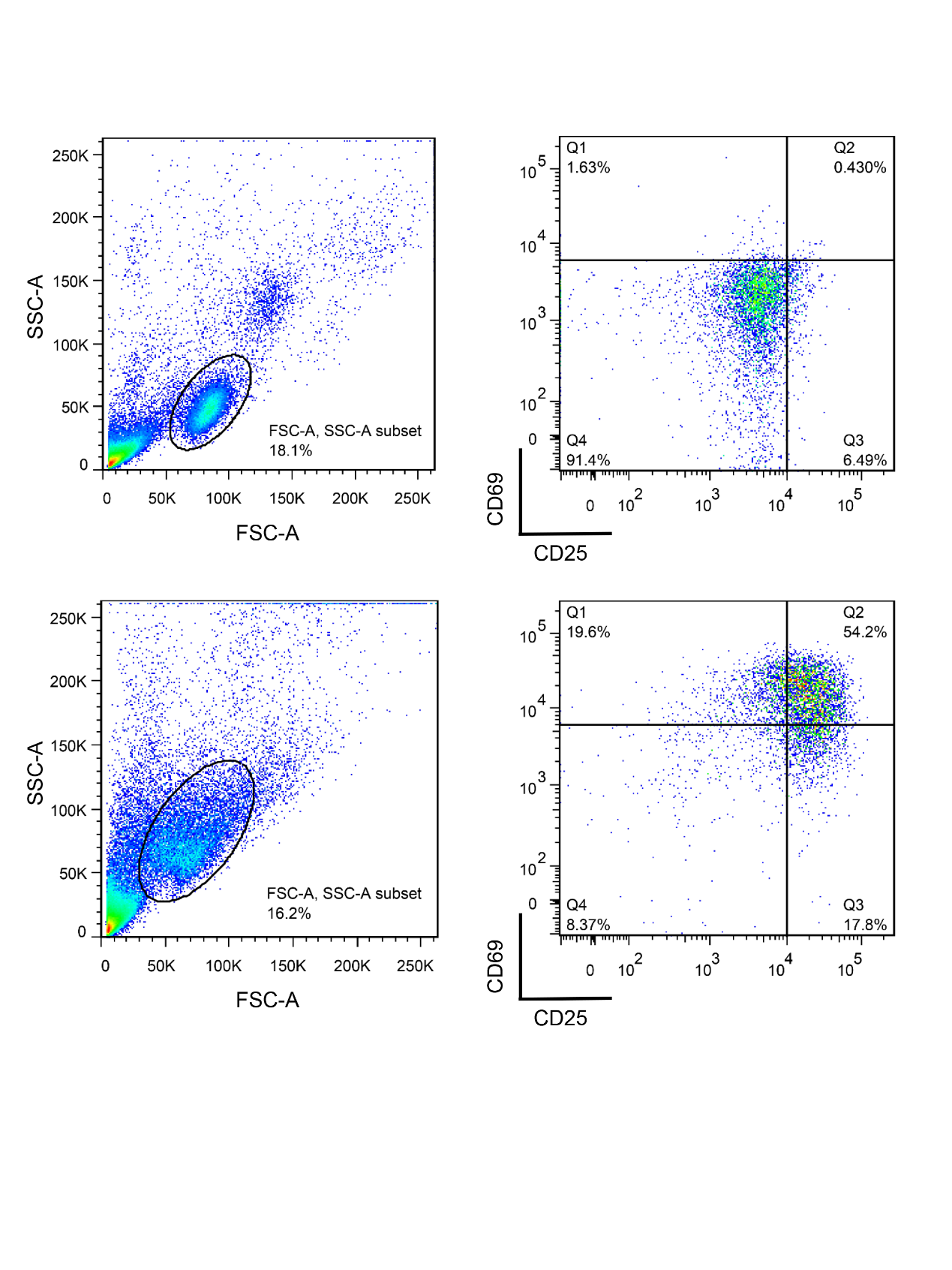
**Supplementary Figure 1. Isolation and activation of PBMCs.** Expression of CD25 and CD69 before (above) and after (below) T cells were activated with OKT-3 for 24 hours *in vitro*.

**
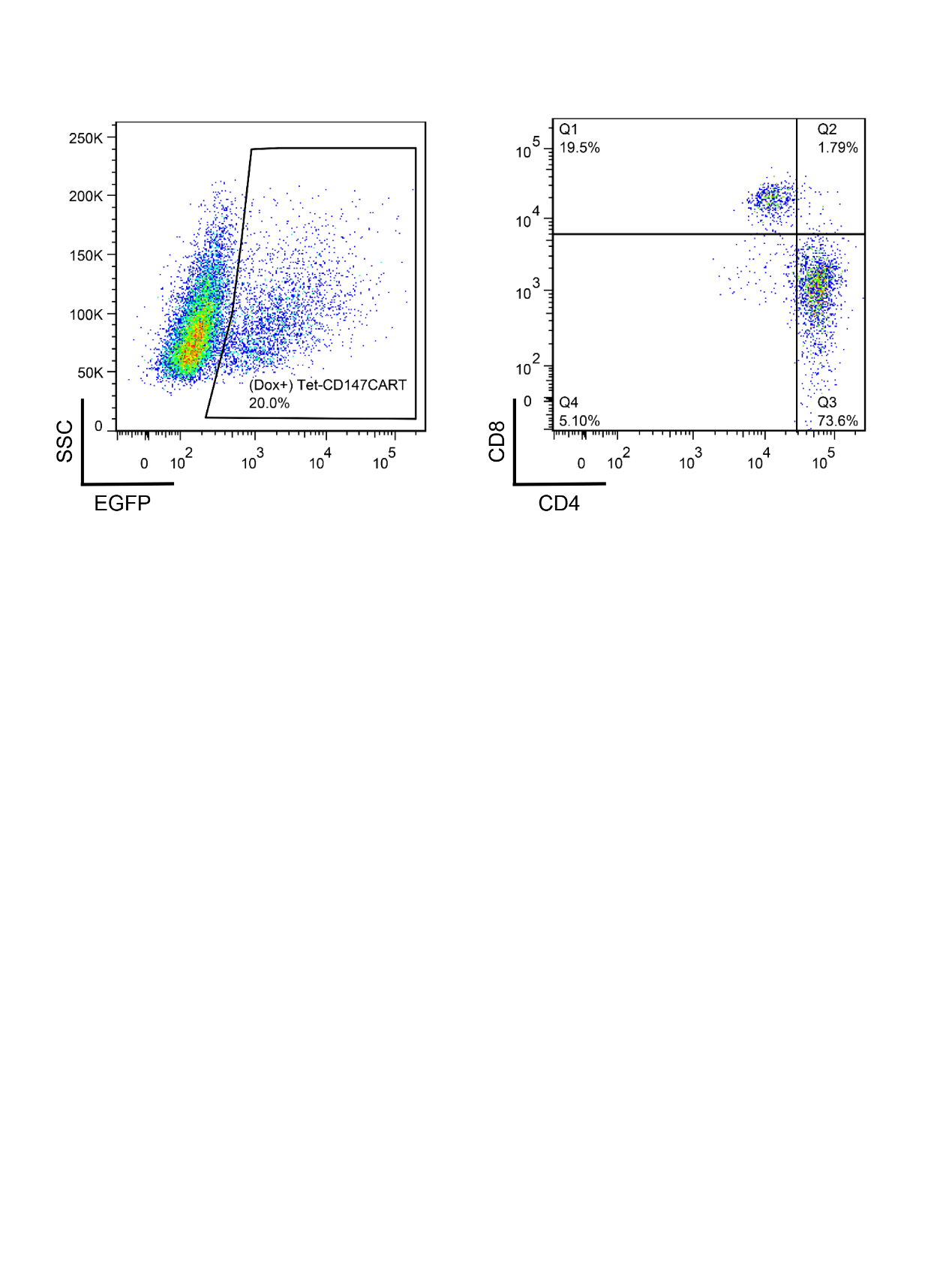
 Supplementary Figure 2. Subpopulation analysis of Tet-CD147CART cells.** Expression of CD4 and CD8 in (Dox+) Tet-CD147CART cells was detected by flow cytometry.


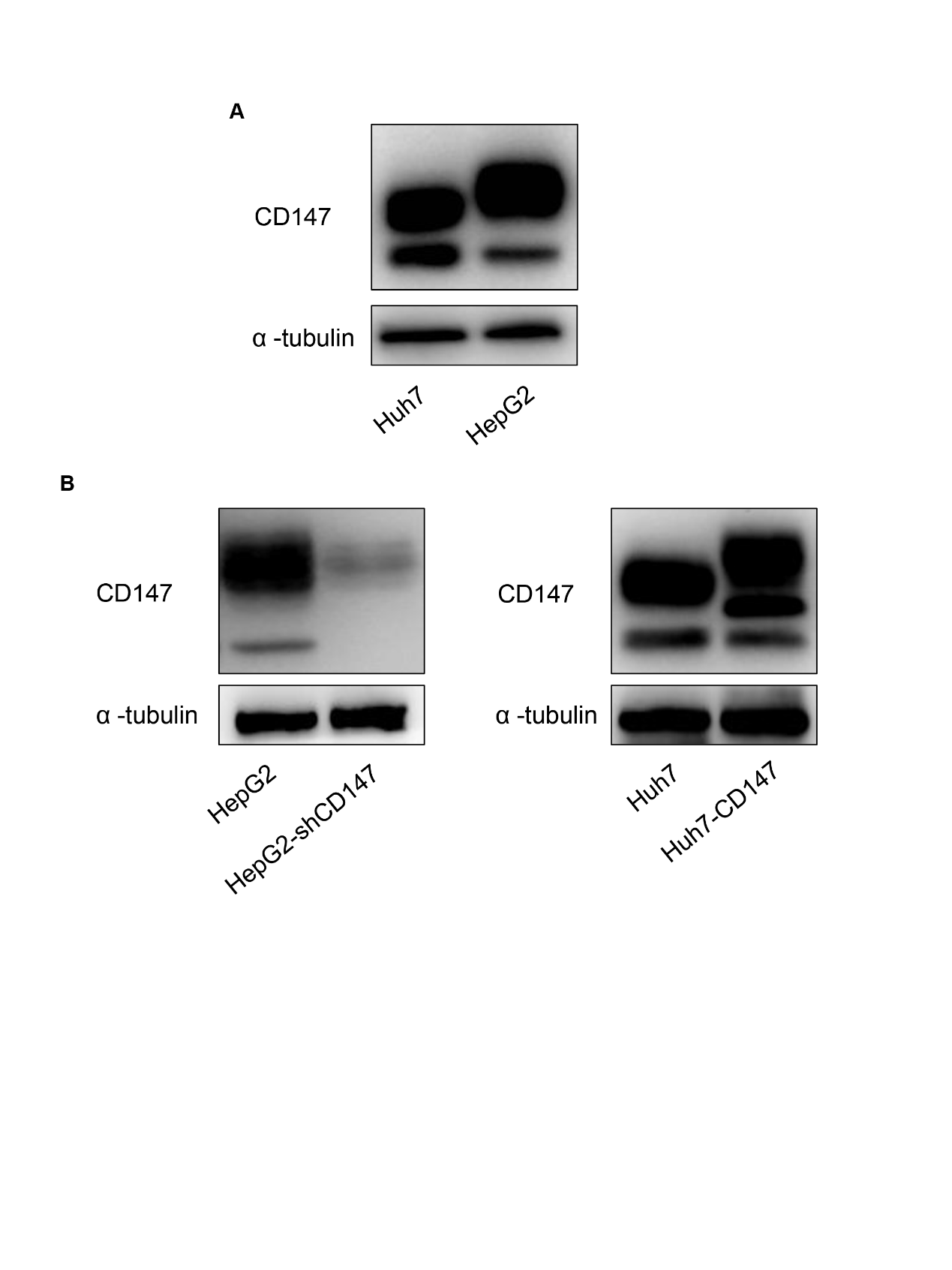


**Supplementary Figure 3. Establishment of stable cell lines.** **(A)** Western blotting analysis of the endogenous expression of CD147 protein in Huh7 cells and HepG2 cells. **(B)** Western blotting analysis of CD147 expression in HepG2 cells with CD147 knockdown (HepG2-shCD147) and Huh-7 cells with CD147 overexpression (Huh7-CD147). Alpha-tubulin was used as a loading control.


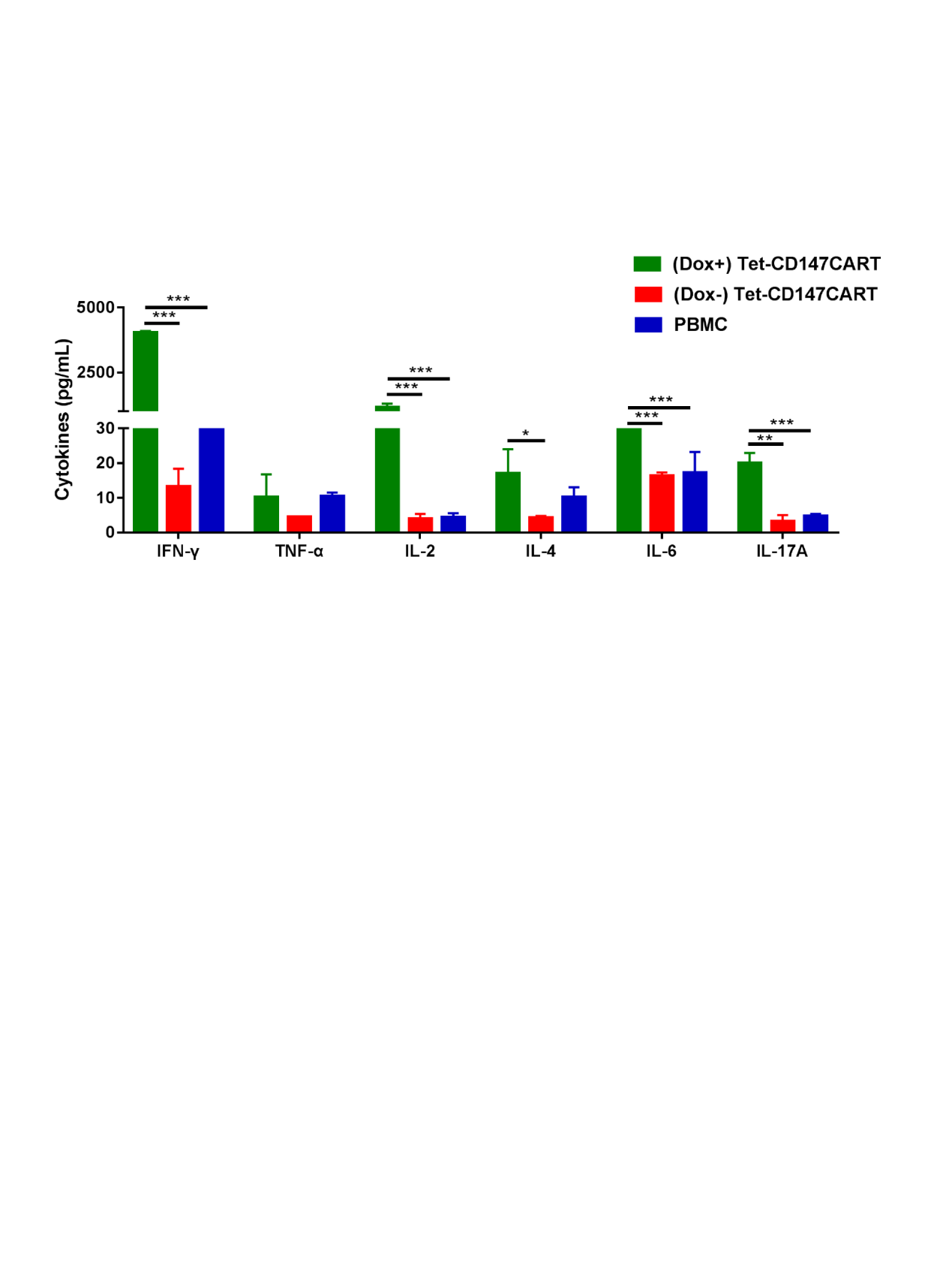


**Supplementary Figure 4. Determination of cytokine secretion.** After (Dox+) Tet-CD147CART cells, (Dox-) Tet-CD147CART cells, and PBMCs co-cultured with Huh-7 cells at an E:T ratio of 10:1 for 16 hours, cytokines IFN-γ, TNF-α, IL-2, IL-4, IL-6, and IL-17A in the supernatants were determined using flow cytometry. n = 3, **P* < 0.05, ***P* < 0.01, ****P* < 0.001.
